# Contributions of Mammalian Chimeras to Pluripotent Stem Cell Research

**DOI:** 10.1016/j.stem.2016.07.018

**Published:** 2016-08-04

**Authors:** Victoria L. Mascetti, Roger A. Pedersen

**Affiliations:** 1 British Heart Foundation Oxbridge Centre for Regenerative Medicine, University of Cambridge, Cambridge, CB2 0SZ, UK; 2 Wellcome Trust-Medical Research Council Cambridge Stem Cell Institute, University of Cambridge, Cambridge, CB2 0SZ, UK; 3 Department of Paediatrics, University of Cambridge, Cambridge, CB2 0SZ, UK

## Abstract

Chimeras are widely acknowledged as the gold standard for assessing stem cell pluripotency, based on their capacity to test donor cell lineage potential in the context of an organized, normally developing tissue. Experimental chimeras provide key insights into mammalian developmental mechanisms and offer a resource for interrogating the fate potential of various pluripotent stem cell states. We highlight the applications and current limitations presented by intra- and inter-species chimeras and consider their future contribution to the stem cell field. Despite the technical and ethical demands of experimental chimeras, including human-interspecies chimeras, they are a provocative resource for achieving regenerative medicine goals.

## Teaser

Experimental chimeras provide key insights into mammalian developmental mechanisms and offer a resource for interrogating the fate potential of various pluripotent stem cell states. Mascetti and Pedersen highlight the applications and current limitations presented by intra- and inter-species chimeras and consider their future contribution to the stem cell field.

## Main Text

### Introduction

Experimental chimeras are widely recognized as the most stringent assays for validating stem cell pluripotency. Preimplantation chimeras provide donor cells with developmental access to the entire fetus and extraembryonic mesoderm (yolk sac, allantois, and amniotic mesoderm), thereby enabling a broad assessment of donor cell developmental capacity. Tetraploid preimplantation chimeras in particular are considered the most comprehensive test of pluripotency because wholly stem cell-derived mouse offspring are the assessment endpoint.

The inner cell mass-like (ICM-like) “naive” mouse embryonic stem cells (mESCs) adhere to the most stringent definitions of pluripotency in that they contribute to all tissues of the developing body in a preimplantation chimera assay including the germline (Bradley et al., 1984, Nagy et al., 1993). Mouse pluripotent stem cells (PSCs) generated by reprogramming of somatic cells either by somatic cell nuclear transfer into nuclear transfer embryonic stem cells (ntESCs) (Munsie et al., 2000, Kawase et al., 2000) or by direct reprogramming into mouse induced PSCs (miPSCs) (Takahashi and Yamanaka, 2006) also share the defining feature of mESCs: they have generated mice wholly derived from donor stem cells following tetraploid complementation (Boland et al., 2009, Lin et al., 2010).

Recently, chimera assays have been more broadly applied to test the lineage potential of other mammalian pluripotent states. Interestingly, epithelial epiblast-like “primed” PSCs (including mEpiSCs, hESCs, and hiPSCs), unlike their ICM-like counterparts (mESCs, ntESCs, and miPSCs), are barely able to form preimplantation chimeras (James et al., 2006, Brons et al., 2007, Tesar et al., 2007, Masaki et al., 2015, Chen et al., 2015). Efforts continue to assess the potential of naive human cells to form preimplantation interspecies chimeras (Gafni et al., 2013, Theunissen et al., 2014, Takashima et al., 2014, Theunissen et al., 2016). Conversely, epithelial epiblast-like PSCs, which resemble the post-implantation epiblast, instead form post-implantation chimeras (Huang et al., 2012, Kojima et al., 2014, Mascetti and Pedersen, 2016).

In this Perspective we focus on the contribution of mammalian chimeras for assessing the competence of PSCs and their respective stem cell states to participate in normal in vivo development. We also consider the lessons gleaned from the embryo's own resident PSCs and how this can inform the in vitro capture of mammalian pluripotent states.

### Definitions of Chimeras

A chimera is a composite organism in which the different cell populations are derived from more than one fertilized egg, thereby combining tissues with distinct genetic origins and identities (McLaren, 1976). The distinct biological mechanisms underpinning chimera formation begin with the persistence of donor cells after transplantation and continue via their participation in the morphogenetic movements of the host embryo, culminating in donor cell differentiation in a manner paralleling the tissue in which they reside.

A primary, or embryonic, chimera is one in which the genetically different cell populations co-exist from a very early stage of embryogenesis, even from fertilization (McLaren, 1976). In light of current and advancing technologies it is pertinent to state that a primary chimera is one in which both host and donor have not undergone organogenesis and thus are capable of contributing to most or all major building blocks of the body. Typically, experimental primary chimeras are formed by combining isolated blastomeres from a minimum of two embryos, by the aggregation of two or more whole early cleaving embryos, or by stem cell transplantation under the zona pellucida or into the blastocyst cavity of a preimplantation embryo. Primary chimera formation, generated by cell transplantation (whether embryo-derived or in vitro-derived stem cells) to the embryo, provides a stringent assessment of stem cell pluripotency.

By contrast, a secondary chimera is one in which tissues are combined from two or more adult individuals, or from embryos after the period of organogenesis has begun (McLaren, 1976). As a consequence of being initiated at a later developmental stage, secondary chimerism is typically limited to one or more tissue-specific lineages.

### A Brief History of Experimental Chimeras

**Figure 1:**
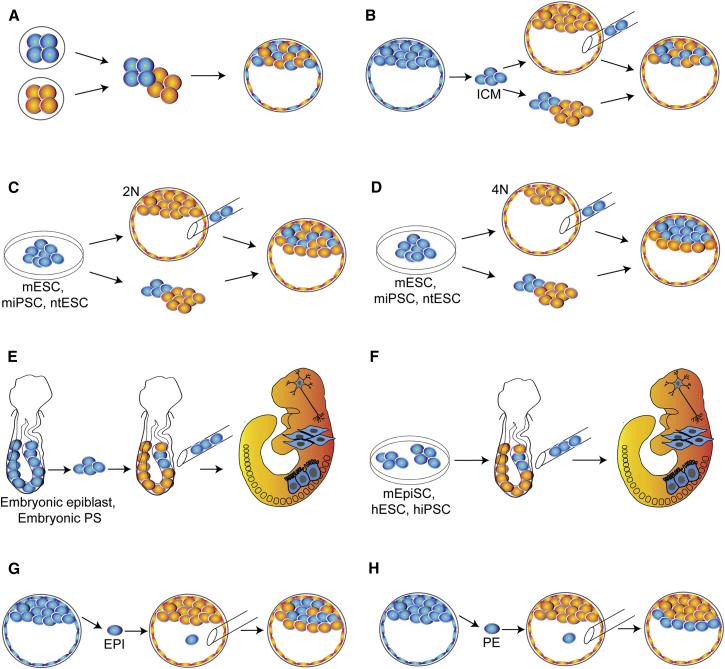
Chimeras: Assays for Mammalian Embryology and Stem Cell Biology Donor cell and donor chimera contribution are depicted in blue; host cell and host chimera contribution are depicted in orange. At the early blastocyst stage (E3.5), the mouse embryo consists of a compact inner cell mass (ICM) and an outer layer of trophectoderm (TE). At the late blastocyst stage (E4.0-4.5), the ICM consists of two morphologically distinct cell populations: the epiblast (EPI) and a layer of primitive endoderm (PE). (A) Whole-embryo aggregation gives donor and host embryo contribution to both inner cell mass (ICM) and trophectoderm (TE) lineages at E3.5. (B) ICM injection into blastocyst, or ICM <-> morula aggregation gives donor contribution to ICM, but not to TE at E3.5. (C) Mouse PSCs injected into blastocyst or aggregated with diploid host embryo (2N) contribute to epiblast, but not primitive endoderm at E4.5. (D) Mouse PSCs injected into blastocyst or aggregated with tetraploid (4N) host embryo contribute to epiblast lineage, while host contributes to PE and trophectoderm at E4.5. (E) Embryonic epiblast or primitive streak (PS) transplanted into post-implantation embryo contributes to three primary germ layers (endoderm, ectoderm, and mesoderm) (F) Mouse EpiSCs, hESCs, or hiPSCs transplanted into post-implantation embryo contributes to three primary germ layers. (G) Single E3.5 or E4.5 epiblast (EPI) cells injected into blastocyst contribute to Epi but not PE at E4.5. (H) Single E3.5 or E4.5 primitive endoderm (PE) cells injected into blastocyst contribute to PE but not EPI at E4.5. See Figure 2 for additional details of lineage contribution by donor and host cells in chimera assays.

**Figure 2:**
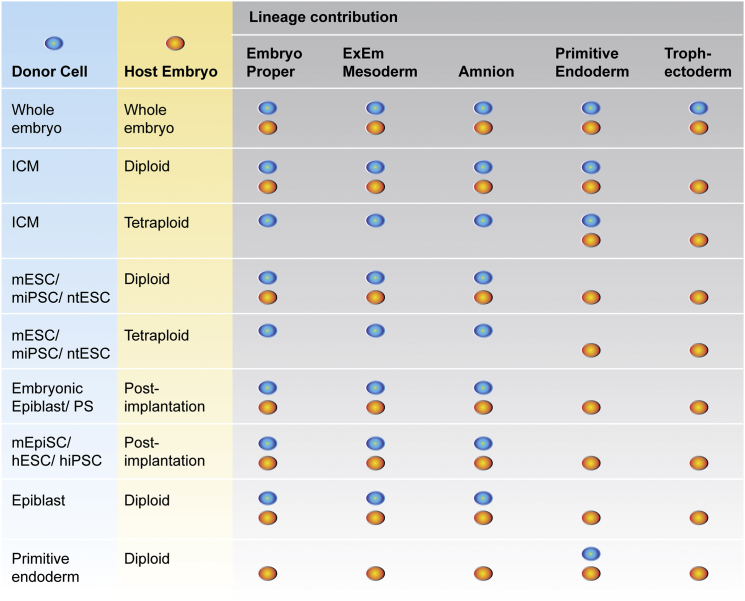
Lineage Contributions of Donor and Host Cells in Chimera Assays Lineage contribution of donor and host cells in chimera assays depicted in Figure 1. ICM includes the epiblast and primitive endoderm. Epiblast-derived tissues include the entire fetus (Embryo proper), plus extraembryonic mesoderm (ExEm Mesoderm) and amnion.

Initially, chimeric potential was assessed by full-term gestation in utero resulting in the birth of offspring: Tarkowski’s pioneering study revealed the capacity for two cleavage-stage embryos to aggregate and form a single chimeric blastocyst (Figure 1A and Figure 2) and for these to develop subsequently to mid- and full-term when transferred to the uteri of foster mothers (Tarkowski, 1961). These primary chimeras resulted in normal-sized mice termed “quadriparental or allophenic” by Mintz (Mintz, 1965), and they were composed of a mixture of cells derived from the two parental embryos (McLaren and Bowman, 1969). Chimerism in such embryos extends throughout both embryonic and extraembryonic lineages, including derivatives of the epiblast, trophectoderm, and primitive endoderm.

Later, chimeras were generated with embryonic cells via the technically challenging procedure of direct injection into the cavity of the host blastocyst (Gardner, 1968) (Figure 1B and Figure 2). After this discovery, mESCs were injected into mouse blastocysts by Evans and co-workers, who reported that mESCs were able to integrate and differentiate into all tissue types in the chimera, including those contributing to the germline (Bradley et al., 1984, Robertson et al., 1986) (Figure 1C and Figure 2).

The developmental potential of mESCs was assessed in parallel with 3.5 day ICM after aggregation with normal diploid (2N) embryos (Figure 1C and Figure 2) or with developmentally compromised tetraploid (4N) embryos (Figure 1D and Figure 2), with both donor types capable of colonizing somatic tissues (Nagy et al., 1990, Nagy et al., 1993). Tarkowski and co-workers first showed that tetraploid embryos demonstrate abortive development (failing between E7.5 and E14) (Tarkowski et al., 1977), but their development can be rescued by complementation with normal diploid embryos to create tetraploid-diploid chimeras. Interestingly, in chimeras made using tetraploid host embryos and diploid embryo, ICM, or mESCs, the resulting epiblast-derived tissues at E13.5 (yolk sac mesoderm, amnion, and fetus) and in newborn mice were derived completely from their diploid component (either embryo, ICM, or cultured mESCs) (Kaufman and Webb, 1990, Nagy et al., 1990, Nagy et al., 1993). However, the yolk sac endoderm and placenta (trophectoderm) lineages were of complete tetraploid origin. Taken together, these pioneering studies provided evidence that mESCs were able to support complete fetal development, and they established tetraploid complementation as an assessment of stem cell pluripotency.

Building on these findings, scientists mutated genes in ESC lines by homologous recombination and transplanted these cells into mouse embryos in order to achieve targeted mutagenesis in the mouse (Doetschman et al., 1988, Thomas and Capecchi, 1990), birthing a revolution in genetic manipulation of mammalian models. The adoption of gene-targeted ESC lines (Rajewsky et al., 1996, Danielian et al., 1998, Shalem et al., 2015) expanded the utility of chimeras, especially when combined with tetraploid complementation (Seibler et al., 2003), to discern gene-development interactions (function and dysfunction) in testing lineage potency and disease modeling. The age of designer mice was conceived.

Post-implantation mouse embryos have been utilized in experimental biology routinely since the 1970s, when New developed a method in Cambridge for culturing rat and mouse embryos (Sadler and New, 1981). The post-implantation mouse embryo opens a window in developmental time, gastrulation, that would otherwise be inaccessible in other mammals (most notably humans) due to practical and ethical challenges. Accordingly, use of post-implantation mouse embryos as chimeric hosts has enabled the assessment of potency and fate of primitive streak (Kinder et al., 1999), epiblast (Tam and Zhou, 1996), and early mesoderm (Parameswaran and Tam, 1995) (Figure 1E and Figure 2). More recently, the ability to generate post-implantation chimeras by the transplantation of epithelial epiblast-like PSCs (commonly referred to as primed) such as mEpiSCs (Huang et al., 2012, Kojima et al., 2014), hESCs, and hiPSCs (Mascetti and Pedersen, 2016) to the post-implantation mouse embryo has removed the barrier to an in vivo functional validation of primed state pluripotency (Figure 1F and Figure 2). Just as the ICM-like pluripotent state of mESCs benefited from pre-implantation embryo chimerism, now the primed state of hPSCs possesses an assay for experimental assessment of its pluripotency.

### Origin and Fate of Embryonic Tissue Lineages as Revealed by Chimera Studies

Chimera studies have been used to determine the potency and fate of embryonic cell lineages based on the capacity for the embryo's resident PSCs (the epiblast of ICM and post-implantation embryo) to participate in embryonic development (Figure 2).

At the late blastocyst stage (E4.0–4.5), the mouse ICM consists of two morphologically distinct cell populations: a compact mass of epiblast cells enveloped by a layer of primitive endoderm on the blastocoelic surface. In order to understand the origin of the embryonic tissues in the developing fetus, distinct from extraembryonic tissues, chimeras were formed by the injection of either primitive endoderm or epiblast cells (the two populations in the ICM) into genetically distinct mouse blastocysts and were analyzed at late gestation (Gardner and Rossant, 1979). These two donor populations had mutually exclusive descendants, primitive endoderm contributing to extraembryonic tissues (especially visceral yolk sac endoderm), and the pluripotent epiblast contributing to the entire fetus (including definitive, or gut, endoderm) and to yolk sac mesoderm, but not to yolk sac endoderm (EPI: Figure 1G; PE: Figure 1H; and Figure 2). The results demonstrate that differentiation of the ICM into two populations in the late blastocyst is accompanied by the acquisition of distinct cell types, as evidenced by the fate of the cells following transplantation. Interestingly, at the early blastocyst stage (E3.5), lineage tracing and aggregation chimera experiments also showed that the majority of single early ICM cells were already restricted to be either epiblast or primitive endoderm, despite displaying no morphological or positional distinction (EPI: Figure 1G; PE: Figure 1H) (Chazaud et al., 2006).

The pluripotent epiblast of the mouse embryo undergoes major progressive transitions during development. A principal example of epiblast transition occurs at E5.0 when the round, multi-layered epiblast cells of the preimplantation ICM become a single layer of polarized cells forming a pseudostratified epithelium, which is accompanied by a dramatic reorganization of the epiblast at implantation (Gardner and Cockroft, 1998; reviewed in Bedzhov et al., 2014). The chimeric contribution of donor cells from post-implantation stage embryos was also examined by blastocyst injection and reimplantation to maternal recipients. Transplants performed using post-implantation epiblast donor cells from E5.5 and E8 into the preimplantation blastocyst showed embryonic and fetal chimera formation, but with a precipitous decline in chimera frequency as the donor epiblast progressed in developmental stage (E5.5: 10.9%; E8: 1.1%) (Moustafa and Brinster 1972). By comparison, when primitive endoderm cells of E5.5 and E6.5 were transplanted, they contributed exclusively to extraembryonic endoderm (mostly parietal) (E5.5: 78.8%; E6.5: 6.2%) (Gardner, 1982).

By striking contrast to the diminished preimplantation chimera rate, post-implantation fetal chimeras were readily achieved using embryonic epiblast cells from primitive-streak-stage mouse embryos (Tam, 1989, Tam and Zhou, 1996) (Figure 1E and Figure 2). Intriguingly, heterotopic transplants revealed broad epiblast plasticity, whereby their progeny adopted fates typical of their site of transplantation. Transplants to the epiblast region bordering on the extraembryonic ectoderm remarkably contributed to the primordial germ cell (PGC) lineage, even when the epiblast cells originated from the region typically developing into brain. This not only reinforced the evidence for epiblast plasticity from single-cell tracing (Lawson et al., 1991), but it also confirmed the origin of PGCs from the embryonic-extraembryonic border region, as observed in cell lineage tracing studies in intact pre- and early- gastrula stage embryos (Lawson and Hage, 1994). Orthotopic primitive streak transplants gave orderly allocation of mesodermal cells to the extraembryonic and embryonic structures, revealing the fate of different streak stages and sites during mouse gastrulation (Kinder et al., 1999). The fidelity of the fate map obtained using chimeras is confirmed by its similarity to the fate of epiblast cells marked by intracellular injection of intact embryos (Lawson et al., 1991). Taken together, these studies prove that post-implantation epiblast and primitive streak tissues can indeed participate in chimera formation, provided that they are transplanted to post-implantation-stage embryos.

### Use of Chimeras for Validation of Epithelial Epiblast-like Pluripotency

**Figure 3:**
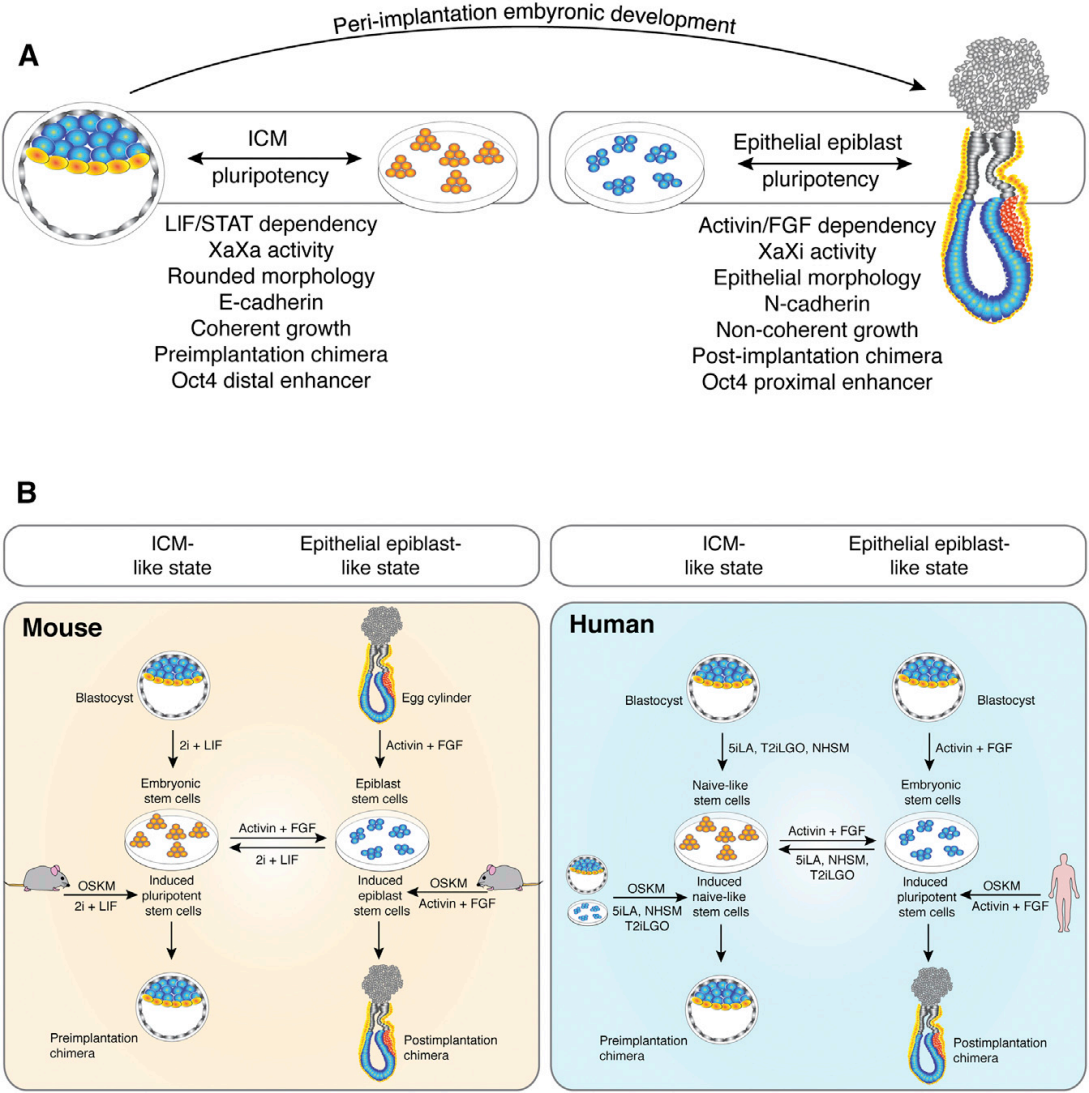
Chimeric Competency of PSC States (A) Properties shared between ICM-like or epithelial epiblast-like PSCs and their respective pluripotent compartments in the embryo. ICM-like mouse and human PSCs share a number of properties with the ICM itself, including signaling dependencies, dual X chromosome activity, cellular morphology and behavior, chimera competency, enhancer usage, and cell surface adhesion. Epithelial epiblast-like mouse and human PSCs likewise share properties with the post-implantation epiblast. The sharing of these and other characteristics can be suggested as the basis for stage-matched chimerism. (B) Assessing pluripotent states by chimera formation. Mouse ESCs and iPSCs, derived from blastocysts and the reprogramming of somatic cells, respectively, have an ICM-like (naive) phenotype and form dome-shaped colonies. mESCs can form chimeras with preimplantation embryos. Mouse EpiSCs, derived from egg cylinder epiblast layer, have an epithelial epiblast-like (primed) phenotype, form flattened colonies, and can form chimeras with post-implantation mouse embryos. mESCs and mEpiSCs can be interconverted through the exchange of their growth media. Human ICM-like (naive) state PSCs derived from blastocysts or by the reprogramming of somatic cells form dome-shaped colonies. Naive hPSCs are expected to form preimplantation chimeras but have done so inefficiently. Human PSCs derived from blastocysts or by the reprogramming of somatic cells in Activin-FGF-containing media have an epithelial epiblast-like (primed) phenotype, form flatted colonies, and can form chimeras with post-implantation mouse embryos. Human naive and primed state hPSCs can be interconverted by having their media conditions exchanged.

A central question regarding the identity of the in vivo embryonic counterpart to PSCs arises from comparison between properties exhibited by human ESCs (and hiPSCs) that distinguish them from mouse ESCs, despite their paralleled derivation from the ICM of the blastocyst. Analysis of the epiblast of the ICM and the epiblast of the post-implantation embryo reveals properties shared between the pluripotent compartments in the embryo and their respective stage-matched PSCs in vitro. These properties may hold the key to unlocking stage-specific chimeric competency (Figure 3A).

In 2007 two groups reported that PSCs could be isolated from the epiblast layer of post-implantation embryos, designated mEpiSCs (Brons et al., 2007, Tesar et al., 2007). The discovery of mEpiSCs provided what some might consider to be the missing piece of the jigsaw puzzle in the field of pluripotency, in revealing a much-needed explanation for the differences between ICM-like mESCs and epithelial epiblast-like hPSCs (Krtolica et al., 2007). mEpiSCs and hPSCs represent a pluripotent state equivalent to the epithelial epiblast layer of gastrulation-stage embryos. It became evident that human PSCs phenocopy mouse epiblast stem cells and thus the epiblast of the egg cylinder (Figure 3A)—this is exemplified by their requirement for Activin and FGF in maintenance of pluripotency (Vallier et al., 2005).

The distinct culture requirements and gene expression programs associated with PSCs captured in vitro likely reflect the dynamic development of the epiblast in the embryo (Kojima et al., 2014, Boroviak et al., 2015) (Figure 3A). In sum, this emphasized that the nature of pluripotency in the embryo changes during development and revealed that different types of PSCs can capture the embryo’s properties as distinct pluripotent states (at present, the ICM-like state and the epithelial epiblast-like state).

A race ensued to capture the missing naive ICM-like state of human pluripotency (reviewed in Hackett and Surani, 2014, Mascetti and Pedersen, 2014, Weinberger et al., 2016, Wu and Izpisua Belmonte, 2015). Between 2010 and 2016, a number of papers emerged reporting the derivation of naive hPSCs comprising hESCs and hiPSCs (Buecker et al., 2010, Chan et al., 2013, Gafni et al., 2013, Guo et al., 2016, Hanna et al., 2010, Takashima et al., 2014, Theunissen et al., 2014, Theunissen et al., 2016, Ware et al., 2014). These intriguing reports detailed various candidate culture conditions to capture the previously missing naive, ICM-like flavor of hPSCs. Nevertheless attempts to generate preimplantation chimeras using naive-like hPSCs have yielded a low rate of chimeras when assessed at fetal stages (Gafni et al., 2013, Theunissen et al., 2014, Theunissen et al., 2016, Takashima et al., 2014, Masaki et al., 2015), often with efficiency too low to be used as an assay for their pluripotency.

Interestingly, mEpiSCs, like both naive and primed state hPSCs (hESCs and hiPSCs), contribute only poorly to preimplantation chimeras, thus providing no information on the functional capacity of the differentiated tissues derived from the stem cells (Brons et al., 2007, Chen et al., 2015, Gafni et al., 2013, James et al., 2006, Masaki et al., 2015, Tesar et al., 2007). Initial speculation led to suggestions that mEpiSCs represent a more restricted (primed) PSC, with some even doubting their pluripotent nature, while mESCs were suggested to symbolize a more developmentally potent (naive) state of pluripotency (reviewed in Nichols and Smith, 2009). Others suggested that the inability of mEpiSCs to form preimplantation chimeras was due to their failure to contribute to the vital extraembryonic tissues, which were speculated to be required for subsequent development into the lineages of the embryo proper (Polejaeva and Mitalipov, 2013). However, a study by Schoeler and co-workers (Han et al., 2010) demonstrated that a specific transgenic mEpiSC line (GOF 18) was more capable of contributing to blastocyst chimeras, albeit at low efficiency, owing to their relative immaturity as compared to other mEpiSC lines.

In 2012, Wilson reported that multiple mEpiSC lines were broadly chimera competent when transplanted to the epiblast of the post-implantation mouse embryo (Huang et al., 2012, Kojima et al., 2014); conversely, mESCs formed teratoma-like clumps, indicating an absence of post-implantation chimeric competency (Huang et al., 2012). In a similar approach, other types of PSCs, such as region-selective hESCs, can also be aligned with their in vivo counterpart by chimera formation. In this manner, region-selective cells showed chimeric contribution when transplanted to the posterior epiblast of the late gastrula-stage mouse embryo, confirming their spatially defined nature as revealed by transcriptional profiling (Wu et al., 2015). Notably, Mascetti and Pedersen transplanted hESCs and hiPSCs to the epiblast and primitive streak of the gastrulating post-implantation mouse embryo and generated interspecies human-mouse chimeras with high efficiency (Mascetti and Pedersen, 2016). Chimeric hPSC progeny showed widespread dispersion and proliferation in the host fetus, as well as differentiation capacity in a manner that paralleled the resident epiblast, thus validating hPSC pluripotency (Mascetti and Pedersen, 2016).

As commonly accepted, evidence for hPSC pluripotency from in vitro differentiation and teratoma assays demonstrates three germ layer competency and has been used as the standard criteria with which to grant pluripotent status to hiPSCs, hESCs, and mEpiSCs. Post-implantation chimeras transcend in vitro differentiation and teratoma assays for assessment of stem cell pluripotency by providing evidence for the capacity of PSCs to participate in normal tissue development and cell fate acquisition in an embryonic context. Post-implantation chimeras, however, do not permit development to term because reimplantation to the mother after post-implantation dissection from the parietal yolk sac is not technically feasible (Beddington, 1985), providing instead a short-term in vitro assay (2–3 days). As development of transplanted cells within recipient embryos can only be tracked within a relatively narrow timeframe, the differentiation potency of hPSCs has not been tested for every tissue lineage (e.g., the germline cells and more advanced germ layer derivatives). Indeed, global contributions would not be expected from gastrula-stage transplants, paralleling orthotopic epiblast and primitive streak transplants (Tam and Zhou, 1996, Kinder et al., 1999), and would only arise from the transplantation of PSCs into preimplantation-stage embryos; however, post-implantation chimeras do provide donor cell access to the entire fetus and extraembryonic mesoderm. Accordingly, both post-implantation and preimplantation chimeras represent primary chimeras enabling validation of stem cell pluripotency, epithelial epiblast-like and ICM-like, respectively (Figure 3B).

### Insight from Intraspecies Chimeras

Chimeric animals have been generated using cleavage-stage blastomeres or ICM in a number of mammalian species besides mice, including sheep (Tucker et al., 1974), rats (Mayer and Fritz, 1974), rabbits (Gardner and Munro, 1974), cattle (Brem et al., 1984), pigs (Brem et al., 1984, Matsunari et al., 2013), and non-human primates (Tachibana et al., 2012). The ubiquity of chimera formation using embryonic blastomeres or whole preimplantation embryos demonstrates that chimera-forming capacity is a general trait of early mammalian embryos. Interestingly, although the derivation of ESCs or ESC-like cells has been reported for other species, only mouse, rat, and pig PSCs have been reported to contribute to chimeras capable of full-term development and germline contribution (Bradley et al., 1984, Li et al., 2008, West et al., 2011). The block to germline chimerism using PSCs in other species may therefore be attributable to the limitations of the PSC lines (or states) derived in those species. This insight is important in interpreting the outcomes of interspecies chimerism experiments.

### Generation of Embryonic Interspecies Chimeras

The amazing degree of variation found between mammalian reproductive strategies is coupled with heterochrony within their early developmental events, differences in the timing of implantation, and diversity in modes of placentation (Wimsatt, 1975). This makes the identification of shared developmental events fundamental to mammalian pluripotency a major challenge. Knowledge about the nature of conserved developmental mechanisms can be gained through interspecies chimeras.

The first interspecies chimeras were generated by Gardner and Johnson, 1973, Gardner and Johnson, 1975), who made mouse-rat chimeras by injection of ICMs into blastocysts; by Mystkowska (1975), who made mouse-vole chimeras; and by Rossant (1976), who aggregated rat ICMs with mouse morulae. Development of the resulting chimeras was abortive as they did not progress beyond early post-implantation stages. Rossant then generated chimeras between embryos of two mouse species, *M. musculus* and *M. caroli*, by ICM injection into blastocysts and by aggregation of eight-cell embryos (Rossant and Frels, 1980, Rossant et al., 1982, Rossant et al., 1983). These developed to term and beyond as mixed-species adults, provided that the chimera had trophoblast cells with a genotype matching that of the foster mother. Subsequent experiments by Fehilly and co-workers generated goat-sheep chimeras by embryo-blastomere aggregation or blastocyst injection (Fehilly et al., 1984), which survived to term and postnatally, again providing that their trophoblast genotype matched the recipient (sheep) mother. Interestingly, the chimeric coat phenotype was mixed, seemingly representing domains of goat and sheep hair in alternating stripes (Fehilly et al., 1984). It is tempting to speculate that relative evolutionary proximity is responsible for the success of Rossant’s interspecific mouse chimeras (7.8 Mya divergence; Time Tree of Life [TToL] [Hedges et al., 2015]) and Fehilly’s goat-sheep chimeras (10.1 Mya; TToL).

### A Chimeric Index for PSC Interspecies Chimeras

The chimera-forming ability of PSCs has been evaluated in vivo by the transfer of injected embryos to uteri of foster mice, where they have been assessed at mid-gestation (fetal) stages and at full term (neonate). Additionally, the chimera-forming capacity of most PSCs has been tested in an in vitro model of integration, where persistence of donor cells was scored at successive days in vitro (DIV) following preimplantation or post-implantation embryo injection. The chimeric index derived from the ratio of these two outcomes (in vitro outcomes/in vivo fetal and full-term outcomes combined = “chimeric index”) gives a measure of the chimera-forming ability of PSC types, whereby the chimeric index should tend to 1 (Table 1, Table S1). As such, the chimeric index can then be used in two ways: (1) as a criterion for chimeric competency, whereby PSCs transplanted into an interspecies host embryo should meet a comparable chimeric index to the intraspecies chimera; (2) to estimate the chimera-forming ability of other PSC cell types that are not amenable to in vivo chimera formation, due to ethical and technical limitations, based on their in vitro chimera outcomes.

As expected for the “gold standard” intraspecific positive control, mESCs show high incidences of full-term chimerism (∼77%) and fetal chimerism (70%); they also showed high levels of contribution to in vitro chimeras (83.5% at 4 DIV), giving a high chimeric index of 1.16 at 4 DIV (Masaki et al., 2015). Similar to mouse ESCs, mouse iPSCs gave high levels of full-term chimerism (60.25%) and fetal chimerism (76%) (Masaki et al., 2015). Subsequent successful efforts to generate rat-mouse chimeras using blastocyst injection of ESCs (Kobayashi et al., 2010, Isotani et al., 2011) indicate that even the more distantly related rat-mouse pair (22.6 Mya, TToL) was capable of chimera formation. When injected into mouse embryos, rat ESCs and iPSCs gave moderate levels of full-term chimerism (rat ESCs, 47%; rat iPSCs, 41%) and gave low levels of in vitro chimeras (rat ESCs, 32%; rat iPSCs, 48.25% at 4 DIV), for a chimeric index of 0.8 and 1.18, respectively (Masaki et al., 2015). Indeed, the rat-mouse species pairing has been recently used to demonstrate organ-specific contribution in interspecies chimeras (Usui et al., 2012, Kobayashi et al., 2010, Isotani et al., 2011). Monkey iPSCs, when injected into mouse preimplantation embryos, were non-chimeric at mid-gestation fetal stages (Fang et al., 2014). However, naive monkey ESCs were able to form fetal chimeras (14.3%) and preimplantation chimeric outgrowths in vitro (51.8% at 2 DIV), (Chen et al., 2015), for a chimeric index of 3.62, which is likely artificially inflated due to the short period of in vitro growth. Mouse EpiSCs, when injected into mouse preimplantation embryos, were scarcely chimeric at full term (0%–1%) (Brons et al., 2007, Tesar et al., 2007), and they gave no contribution to in vitro implantation (Masaki et al., 2015), for a chimeric index of 0. Strikingly, however, when mEpiSCs were transplanted to the post-implantation mouse embryo, they gave high levels of chimerism (≥80% after 1 or 2 days culture) (Huang et al., 2012). Brivanlou and co-workers injected hESCs into mouse blastocysts or aggregated them with cleaving mouse embryos and examined the resulting embryos for chimerism both in vitro and after transfer to foster mothers for 5 days of gestation (E8) (James et al., 2006). The low rate of normally developing fetal chimeras (4.17%) that resulted had sparse hESC contribution (an illustrated embryo had only 10 human cells) (James et al., 2006), and this, together with the complete lack of hiPSC persistence in an in vitro chimera model (Masaki et al., 2015), could be interpreted to mean that distantly related species such as human and non-human primates (90.9 Mya, TToL) are not in fact capable of robust chimera formation with preimplantation mouse embryos. Indeed, the low rate of fetal chimera formation observed when human naive-like PSCs were injected into mouse morula/blastocyst (6.19% and 0.9%, depending on the study) (Gafni et al., 2013, Theunissen et al., 2016) suggests that further work will be required to fully address the capacity of naive-like hPSCs to form robust preimplantation chimeras in mice. In striking contrast, when hESCs and hiPSCs were transplanted to post-implantation mouse embryos, they resulted in high rates of chimera formation (99.2% and 71.8%, respectively), accompanied with robust and widespread dispersion and proliferation of graft-derived progeny (Mascetti and Pedersen, 2016). Correspondingly, region-selective hESCs form chimeras (60.69%) when transplanted to their matched location in the post-implantation mouse embryo.

It is entirely plausible that any cell types showing a lower chimeric efficiency than documented for the intraspecies control assay in vivo or in vitro (Table 1) may be considered less chimera competent. Differences in chimera competence may be traced to a variety of alternative mechanisms such as (1) the inhospitable nature of the host embryo, (2) species-specific differences in ESC maintenance and culture, which pose challenging factors in the identification and derivation of putative ESCs, and (3) a block to interspecies chimerism, or more specifically a developmental-stage-specific interspecies barrier, which would require the precise matching of the developmental window for each species in order to be overcome.

### Parameters for Effective Chimera Formation

Segregation of the early lineages in the developing embryo, the epiblast and the primitive endoderm in the ICM, may be a determinative mechanism in the stage-matching of host and donor cell to form chimeras. At peri-implantation in the mouse, when segregation is morphologically evident, there is a significant decline in the ability of the host ICM to incorporate donor cells and form mouse chimeras (Ohta et al., 2008). Interestingly, analysis of marmoset preimplantation blastocysts revealed that their ICMs had already segregated into clusters of NANOG-positive epiblast covered by GATA-6-positive primitive endoderm cells (Boroviak et al., 2015). As such, we suggest that segregation might prevent incorporation of transplanted donor cells into the host ICM. In support of this hypothesis, and fascinatingly, monkey chimeras were efficiently generated by aggregating cleaving four-cell embryos, before the ICM is evident (Tachibana et al., 2012).

It is important to emphasize the pivotal role PSC states seem to play in unlocking chimeric competency. Human and monkey PSCs are derived and maintained in an epithelial epiblast-like state, which has very distinct biological properties from the ICM (Figure 3A). These differences, by analogy with mEpiSCs, may importantly include altered expression of intercellular adhesion molecules (Ohtsuka et al., 2012) and an epithelial rather than globular cellular morphology (Gardner and Cockroft, 1998). Accordingly, we have to consider the epithelial epiblast-like state of hPSCs and mEpiSCs as an alternative explanation for their lack of chimera-forming ability observed to date with mouse preimplantation embryos. This conclusion is seemingly supported by Nakauchi and co-workers’ findings that rat and mouse ESCs and iPSCs formed full-term in vivo chimeras efficiently with mouse blastocysts, but monkey ESCs did not persist in vivo to E8.5 (Masaki et al., 2015). In a similar vein, ICM-like mESCs do not incorporate into the post-implantation mouse embryo, instead forming teratoma-like clumps that express OCT4 and other pluripotency-associated factors (Huang et al., 2012). The existence of PSCs in distinct developmental pluripotent states therefore leads to the conclusion that stage-matching ICM-like PSCs to the preimplantation embryo and epithelial epiblast-like PSCs to the post-implantation embryo may hold the key to unlocking efficient chimera formation (Figure 3B).

Interestingly, current evidence indicates only scant ability of naïve-state hPSCs to incorporate into mouse morulae/blastocysts, to which they are presumably stage matched (Gafni et al., 2013, Theunissen et al., 2014, Theunissen et al., 2016, Takashima et al., 2014, Masaki et al., 2015). Indeed, alternative conditions for defining naive human PSCs may result in a differing alignment of stem cell stage when compared to the human embryo, and, in turn, diversity in their chimeric competency. In the most recent report of naive human pluripotency, Theunissen et al. concluded that 5i/L/A-maintained naive hESCs are closely related to the late-morula and early blastocyst-stage human embryo using their highly sensitive transposon transcription signature method. Significantly, now that the absence of a human-mouse interspecies barrier has been demonstrated (Mascetti and Pedersen, 2016, Wu et al., 2015), the sporadic integration of naïve-state human stem cells into the preimplantation embryo may call into question their putative naive pluripotent state.

Developmental normality is an inherent technical challenge in generating chimeras. As the host embryo species seems to dictate many of the resulting chimera’s characteristics, including its size (Kobayashi et al., 2010), it is pivotal to ensure that in vitro development of the host embryo parallels that observed in utero. Indeed, the question remains, what extent of donor cell contribution is required to achieve a bona fide chimera? The idealized stereotype, equal contribution based on having two parental origins, is an oversimplification of the biological process of development. However, many biological factors will influence the extent of donor cell contribution, including the rate of cell division, which differs with stage of development, transplant location, and donor cell incorporation efficiency. Additionally, host-donor competition can dramatically affect the extent of donor cell contribution. Moreover, stochastic mechanisms can enable some stem cells to have more descendants than others such that gradually one source will dominate (Krieger and Simons, 2015). The tendency of one source to prevail may result from division orientation or other still poorly understood mechanisms that enable some stem cells to have more descendants than others (Krieger and Simons, 2015). Similarly, chimeric drift—where the proportion of contribution by each member of the chimera changes over time—may play a role in chimeric contribution, as certain strains have been shown to predominate over others and so may have a demonstrable competitive advantage (Ahmad et al., 1993).

Researchers have sought to increase the extent of graft-derived tissue contribution. To this end, growth-enhancing or proliferative transgenic lines have been used in order to achieve a more prolific chimeric contribution. However, one remains skeptical about the normality and thus use of these chimeras; for example, c-*myc* transgenic chimeras resulted in overgrowth of graft cells and subsequent abnormalities in the chimeric mice likely due to the imbalance of growth potential between the *myc* transgenic and the normal cells in the same chimeric individual (Augustin et al., 1998). Additionally, anti-apoptotic “don’t die” transgenic lines such as BCL-2 may enable donor cells to survive long enough to incorporate into the correct location and stage of the developing host embryo, or cell adhesion molecules may be utilized to enhance engraftment of donor cells and increase chimeric rate (Ohtsuka et al., 2012). While demonstrating that a transgenic cell line has a selective advantage over a normal line (a principle already established in cell cultures), these chimeras will remain under a cloud regarding their normality and may thus have their developmental or clinical utility called into question.

### Dissecting the Ethical Issues of hPSC and Human Embryo Research

**Figure 4:**
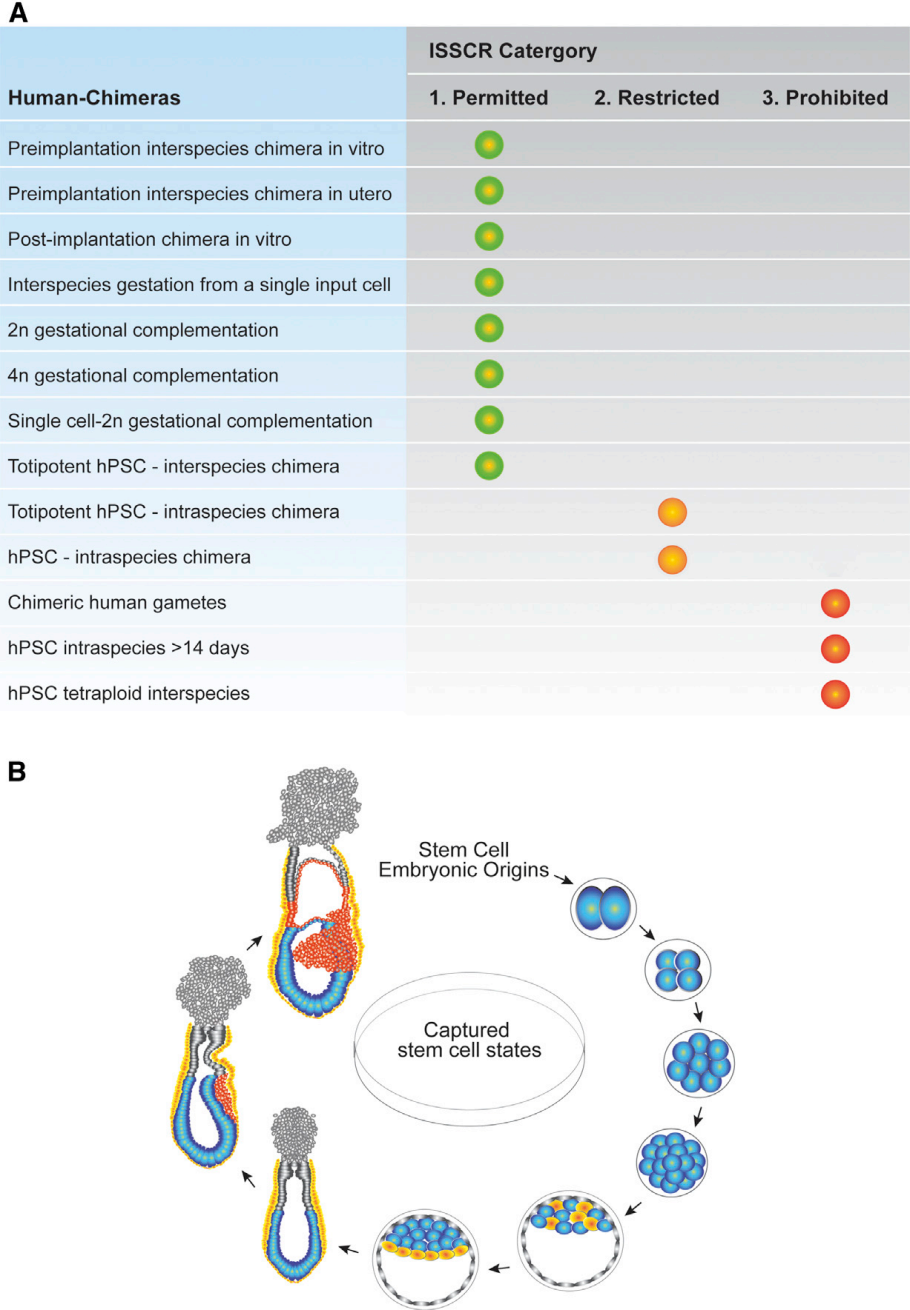
Ethical and Future Perspectives for PSC Chimeras (A) Ethical regulation of human chimera research. Diverse approaches to chimera research involving hPSCs and human embryos are listed, ranked according to their category of review and permissibility as recommended by the ISSCR (ISSCR, 2016). (B) Embryonic origins of stem cells for future in vitro capture. Totipotent and pluripotent stem cell populations in the developing embryo (from two-cell to E7.5), shown in blue, may have the potential to be isolated in vitro as self-renewing stem cell states.

All aspects of hPSC and human embryo research have an ethical dimension. This begins with the patient consent required for derivation of hESCs, which come from surplus human embryos from infertility therapy or patient donation of somatic cells for reprogramming into hiPSCs. New ethical issues have emerged in connection with the generation of interspecies chimeras containing progeny of hPSCs, and these extend into still uncharted territory amidst future prospects for regenerative medicine application of animals containing human material (ACHM) (Figure 4A). Extensive dialog between stem cell researchers and ethicists during the past 2 decades established ethical standards for the experimental use of hESCs (Hyun et al., 2007, Streiffer, 2010). The discovery and adoption of hiPSCs in human stem cell research has led to reduced ethical complexity, owing to their somatic cell provenance. Regardless of source, the use of hPSCs for chimera studies raises additional ethical issues because of their potential for contribution to the regions of the developing fetus that remain controversial (e.g., brain and gonads). Accordingly, academic organizations with responsibility for oversight of stem cell research have promulgated guidelines for experiments that combine hPSCs and mammalian embryos. These specify the categories of research on hPSCs according to their need for oversight (review, approval, and on-going monitoring) by the Embryo Research Oversight (EMRO) process in the parent institute. The International Society for Stem Cell Research (ISSCR) has established and recently updated its recommendations for EMRO review (ISSCR, 2016, Daley et al., 2016), as summarized in Figure 4A. Specifically, these guidelines recommend EMRO review for any experiments involving chimera formation between human totipotent or pluripotent stem cells and human embryos. Moreover, they forbid experiments in which human embryos or embryo-like structures are cultured beyond 14 days or the time of primitive streak formation (whichever comes first). They also forbid experiments in which human-animal chimeras with potential human gametes are bred to each other. Taken together, these constraints on hPSC research leave ample scope for experiments that address major biological questions posed here.

The interest shown by researchers in forming pre-gastrula interspecies chimeras has led the US NIH to suspend funding for research on “pre-gastrulation” (preimplantation) chimeras for a period of review. This action does not seem warranted given the already promulgated ethical parameters. In view of a policy that will likely hinder the great leaps currently being made by researchers, one only hopes that as scientists we continue to work with both regulatory and funding bodies to ensure best practice while also enabling the quest for innovative, elegant, and paradigm-shifting experimental research.

### Perspective for PSC Chimeras in Regenerative Medicine

Chimeras, in particular those using mESCs as the donor cell type, have become a revolutionary experimental tool to study the gene function in knockout models. Impressively, more than 9,000 genes have been targeted in mESCs in the International Knockout Mouse Consortium (Skarnes et al., 2011) and their phenotypes are currently being assessed in large-scale programs (White et al., 2013). Additionally, transgenes, mutations, and gene-targeting in mESCs have been utilized to induce or suppress formation of specific organs/tissues in chimeras (Usui et al., 2012, Kobayashi et al., 2010, Isotani et al., 2011). The elimination of the target organ by knockout of an essential gene (e.g., Pdx1 in pancreas development) in the host embryo creates an open niche for contribution by PSCs of the donor. This approach has been used to generate interspecies mouse-rat chimeras with extensive contribution to specific organs (pancreas [Kobayashi et al., 2010], thymus [Isotani et al., 2011], and kidney [Usui et al., 2012]). Moreover, this strategy is envisaged for use in creating human organs for regenerative medicine through human-animal chimerism (reviewed in Rashid et al., 2014). However, the ultimate realization of this vision will be extremely challenging, owing to the limited human contribution in preimplantation embryo chimeras seen to date and the contribution of host blood vessels to such organs. The generation of human-naive PSC preimplantation chimeras remains a holy grail in this endeavor.

The ethical and xenotransplantation obstacles inherent in generating humanized organs in domestic species make it very challenging. Growing entire organs from embryonic rudiments completely in vitro—recapitulating the process of organ formation and growth—seems equally if not more difficult. An alternative to generating a whole organ may be to generate the functional subunits of an organ in vitro (Pedersen et al., 2012, Yin et al., 2016) Most organs are composites of functional subunits (the smallest elements of an organ that retain the essential activity performed by the organ). The use of genetically deficient/rescue chimeric models may revolutionize the generation of organs either by generating organs for transplant or, perhaps more realistically, by enabling the study of human organ generation in vivo in real time. If a synthetic organ requires only the functional role of an organ and not its gross morphology and size, then reconstituting organs in the mouse embryo provides an expedient approach to modeling organ regeneration without the technical and experimental challenges of larger mammalian models.

The progress in achieving functional tissues in vitro is counterbalanced with the lack of information about human embryogenesis, which presents an obstacle in understanding how closely in vitro pluripotency and differentiation of human PSCs mimic normal development. Human-mouse chimeras (pre- and post-implantation) will facilitate the study of early human development including lineage mapping, cell fate decisions, embryonic signaling cascades, and differentiation mechanisms of human-tissue-specific progenitors during normal development, as well as provide a functional validation of human donor cells (PSCs or tissue-specific progenitors). These chimeras, especially post-implantation chimeras where ex vivo development parallels in vivo development, will also enable real-time assessment of in vivo developmental progression; this is particularly relevant where the specific developmental stages are inaccessible in humans (e.g., gastrulation) and when roles of developmental processes such as cell cycle (Boward et al., 2016) and stem cell loss (Krieger and Simons, 2015) are still poorly understood as determinants of differentiation and cell fate specification. Moreover, the advent of CRISPR technology (Seruggia and Montoliu, 2014) and its use not only for the derivation of transgeneic lines but also in direct modification of the embryo genomes will usher in a new phase in functional genomics in experimental chimeras.

### An Embryonic Counterpart for PSCs

Despite our apparent ability during the past 40+ years to capture PSCs in vitro, pluripotency itself remains enigmatic. While PSCs exist only transiently in the developing embryo, when established in vitro as cell lines, PSCs can be maintained and propagated indefinitely using adaptive culture conditions. One could surmise that in vitro pluripotent states capture snapshots in developmental time. To this end, it is important to determine the in vivo counterpart of in-vitro-derived cells (pluripotent states or tissue-specific progenitors) in order to provide a developmental context in which to frame them and thus enable researchers to use the cells in the most efficient manner and to their full potential. This is especially important because, unlike mESCs, hPSCs do not resemble their original starting material, the ICM or somatic cells.

It seems that a realistic goal for future research is to capture distinct PSC identities or states in vitro that resemble the pluripotent epiblast of the embryo at each day of development until such time as the embryo develops an embryonic axis at gastrulation, at which time it would be possible to mimic the regional and positional information in the embryo. The age of single-cell transcriptomics will aid in the dissolution of our embryonic ignorance and, ultimately, the timely capture of alternate pluripotent states in vitro. The resultant cell populations will capture still-elusive pluripotent and even totipotent cell states. As such, it may be possible in the future to isolate stem cell populations resembling totipotent cleavage blastomeres, E3.5 ICM and E5.0 epiblast, and pluripotent states that would capture each axis pole in the gastrulating embryo (anterior, posterior, distal, and proximal). The beauty of this vision is that each state can be functionally validated by chimeric stage-matched transplantation to the developing embryo. In sum, there may be much more to pluripotency in the developing embryo than we currently witness in the dish.

## Figures and Tables

**Table 1:** Summary of Pluripotent Stem Cell Chimera Rates

Pluripotent Donor Cell Types	Host Embryo	In Vivo Chimera Rate	In Vitro Chimera Rate	Chimeric Index	References
Mouse ESC	preimpl. mouse	high fetal, high neonate	high 4 DIV, mod. 5 DIV	1.16 4 DIV, 0.68 5 DIV	Masaki et al., 2015;
Mouse iPSC	preimpl. mouse	high fetal, high neonate	– –	– –	Masaki et al., 2015, Kobayashi et al., 2010, Usui et al., 2012;
Mouse iPSC	preimpl. rat	mod. fetal	–	–	Kobayashi et al., 2010
Mouse EpiSC	preimpl. mouse	none fetal	none 1–5 DIV	0 1–5 DIV	Brons et al., 2007, Tesar et al., 2007, Guo et al., 2009, Han et al., 2010;
Mouse EpiSCs GOF 18	preimpl. mouse	low fetal, low adult	low 1 DIV	1.12 1 DIV	Han et al., 2010
Mouse EpiSCs	post-impl. mouse	–	high 1–2 DIV	–	Huang et al., 2012, Kojima et al., 2014, Tsakiridis et al., 2014, Wu et al., 2015
rs Mouse EpiSCs	post-impl. mouse	–	high 1.5 DIV	–	Wu et al., 2015
Rat ESC	preimpl. rat	high fetal	–	–	Kobayashi et al., 2010
Rat ESC	preimpl. mouse	mod. fetal, mod. neonate, low fetal, low neonate	mod. 4 DIV, low 5 DIV, –, –	0.8 4 DIV, 0.4 5 DIV, –, –	Masaki et al., 2015, Kobayashi et al., 2010, Isotani et al., 2011
Rat iPSC	preimpl. mouse	low neonate, mod. neonate	–, mod. 4 DIV, low 5 DIV	–, 1.18 4 DIV, 0.58 5 DIV	Kobayashi et al., 2010, Masaki et al., 2015
Rat iPSC	preimpl. rat	high fetal	–	–	Kobayashi et al., 2010
Pig iPSC	preimpl. pig	high neonate	–	–	West et al., 2011
Monkey ESC	preimpl. monkey	none fetal	–	–	Tachibana et al., 2012
Monkey ESC	preimpl. mouse	none fetal	low 4 DIV, none 5 DIV	0 5 DIV	Masaki et al., 2015
rs monkey ESCs	post-impl. mouse	–	high 1.5 DIV	–	Wu et al., 2015
Naive monkey iPSC	preimpl. mouse	sporadic fetal	–	–	Fang et al., 2014
Naive monkey iPSC	preimpl. monkey	low fetal	high 2 DIV	3.62 2 DIV	Chen et al., 2015
Monkey iPSC	preimpl. mouse	none fetal	–	–	Fang et al., 2014
Naive human iPSCs	preimpl. mouse	– –	mod. 3 DIV, low 4 DIV, none 5 DIV	– –	Takashima et al., 2014, Masaki et al., 2015
Naive human ESC	preimpl. mouse	positive fetal, none fetal, sporadic fetal	spor. 1 DIV, –, –	0.48 1 DIV, –, –	Gafni et al., 2013, Theunissen et al., 2014, Theunissen et al., 2016
Human ESCs	preimpl. mouse	low fetal	mod. 2 DIV	9.35 2 DIV	James et al., 2006
Human ESCs	post-impl. mouse	–	high 2 DIV	–	Mascetti and Pedersen, 2016
rs Human ESCs	post-impl. mouse	–	high 1.5 DIV	–	Wu et al., 2015
Human iPSC	preimpl. mouse	–	low 4 DIV, none 5 DIV	–	Masaki et al., 2015
Human iPSCs	post-impl. mouse	–	high 2 DIV	–	Mascetti and Pedersen, 2016

Chimeric contribution from diverse donor cell types in fetal, full-term, and in vitro chimera assays. Table 1 summarizes representative chimera contributions for mouse, rat, pig, monkey, and human PSCs (embryonic stem cells, ESCs; induced pluripotent stem cells, iPSCs; and epiblast stem cells, EpiSCs). Outcomes shown include fetal and full-term (neonate) in vivo chimera rate (where available), in vitro chimera rate (showing data for successive days in vitro [DIV]), and the ratio of their respective rates, the “chimeric index” (in vitro/in vivo full-term plus fetal averaged as overall chimeras) where this could be calculated. Outcomes of PSC chimera rate both in vivo or in vitro are shown; high > 50%, moderate < 50%, low < 25%, sporadic < 5%, none = 0%. Table S1 provides additional details on the data summarized in Table 1.

## Supplemental Information

Document S1. Table S1

Document S2. Article plus Supplemental Information
