# Supplementary material for: Contributions of Mammalian Chimeras to Pluripotent Stem Cell Research
Source: Cell Stem Cell. 2016 Aug 4;19(2):163–75. doi: 10.1016/j.stem.2016.07.018 (PMC5366358; doi:10.1016/j.stem.2016.07.018)
Supplement: Document S1. Table S1 [file mmc1.pdf]

Cell Stem Cell, Volume *19*

Supplemental Information

Contributions of Mammalian Chimeras to Pluripotent Stem Cell Research

Victoria L. Mascetti and Roger A. Pedersen

Summary of pluripotent stem cell chimera rates				
Pluripotent donor cell type	*In vivo* chimera rate	*In vitro* chimera rate	Chimeric index (ratio of *in vitro*/ *in vivo* chimera rates)	References
Mouse ESC – Preimplantation mouse embryo	67.8% (40/59) pups	--	--	Tesar et al., 2007
	77% neonate; 70% fetal; 72.3% overall	83.5% 4DIV; 49.25% 5DIV	1.155 (4DIV/overall chimera);<br>0.68 (5DIV/overall chimera)<br>3.9 (1DIV/fetal chimera)	Masaki et al., 2015
	23% (11/47) E13.5 fetal	91% 1DIV	3.96 (1DIV/fetal chimera)	Ohtsuka et al., 2012
	45.8% (11/24) fetal<br>64.3% (18/28) adults	--	--	Kobayashi et al., 2010
	55.6% (5/9) neonates<br>70.6% (24/34) adults	--	--	Usui et al., 2012
Mouse iPSC - Preimplantation mouse embryo	60.25% neonate; 76% fetal; 63.4% overall	--	--	Masaki et al., 2015
	27% (44/163) postnatal	--	--	Okita et al., 2007
	46.2% (12/26) fetal<br>96.6% (28/29) adults	--	--	Kobayashi et al., 2010
	91.9% (34/37) adults	--	--	Usui et al., 2012
Mouse iPSC – Preimplantation rat embryo	25% (6/24) fetal	--	--	Kobayashi et al., 2010
Mouse EpiSC – Preimplantation mouse embryo	0.5% (1/204), E9.5 fetal stage;<br>1.1% (2/181), low level, ~10% contribution at full term; 0.77% (3/385) overall [however, Brons et al. paper text states 2/385, or 0.5% overall]	31% morula aggregation but without proliferation, suggesting, "…they had not become integrated into the ICM in most cases."	0 (1DIV/ overall chimera)	Brons et al., 2007
	0% (0/35) pups of 49 transferred injected blastocysts	0% (0/23) at 24h after morula aggregation	0 (1DIV/ neonate chimera)	Tesar et al., 2007

	0% at E13.5 (0/12 from normal EpiSCs; 0/30 GFP- EpiSCs; 0/42 total EpiSCs at E13.5) No chimeras at full term	0% ICM integration 8h after blastocyst injection	0 (1DIV/ fetal chimera)	Han et al., 2010
	0% (0/7) EpiSC-injected embryos chimeric at E6.5 (Fig S6)	Obvious reduction in fluorescence at 44 h (Fig S6)	0 (2DIV/ embryonic chimera)	Guo et al., 2009
	--	0% 4DIV; 0% at 5DIV	--	Masaki et al., 2015
Mouse EpiSC GOF 18 - Preimplantation mouse embryo	Somatic 8.9% by PCR and gonadal chimerism 4.4% by fluorescence seen from GOF18 Oct4-GFP+ EpiSCs at E13.5; 8.9% adult chimerism (4/45 injected) seen from Oct4-GFP+ EpiSCs	10% ICM integration 8h after blastocyst injection	1.12 (1DIV/ overall chimera)	Han et al., 2010
Mouse EpiSCs - Post-implantation mouse embryo	--	80.4% chimeric after 1 or 2 days (45/56 embryos)	--	Huang et al., 2012
	--	(34 embryos injected)	--	Kojima et al., 2014
	--	98% chimeric after 1 or 2 days (50/51 embryos)	--	Tsakaridis et al., 2014
	--	80% chimeric (24/30)	--	Wu et al., 2015
Region-selective EpiSCs -Post-implantation mouse embryo	--	51% chimeric (25/49 embryos after E7.5 injections)	--	Wu et al., 2015
Reverted mouse EpiSCs (EpiSC-iPS) – Preimplantation mouse embryo	55.8% (19/34) full term (Table S1); 100% (3/3) positive at E6.5 (Fig S6)	89.5% (17/19 shown in Fig S6 are positive) Little reduction in fluorescence at 44 h	1.60 (2DIV/ neonate chimera)	Guo et al., 2009
	58.6% (17/29) full term; 78% (39/50) at E13.5, of which 59% (23/39) had gonadal chimerisms; 87.1% (74/85) at E6.5	--	--	Bao et al., 2009

Mouse EpiSC-subclone (sub) - Preimplantation mouse embryo	38% neonate;<br>29.5% fetal; 33.75% overall chimeras	52.33% 4DIV;<br>28.67% 5DIV	1.55 (4DIV/overall chimeras);<br>0.849 (5DIV/overall chimeras)	Masaki et al., 2015
Mouse EpiSC-E-caderin - Preimplantation mouse embryo	3.92% GFP^+ E13.5 Dox^+<br>0% GFP^+ E13.5 Dox^- (control)	76.15% survival 1DIV<br>65.35% survival 1DIV	19.43 (1DIV/E13.5 chimeras)<br>--	Ohtsuka et al., 2012
Rat ESC-Preimplantation rat embryo	54.5% (18/33) fetal	--	--	Kobayashi et al, 2010
Rat ESC - Preimplantation mouse embryo	47% neonate;<br>33% fetal; 40% overall chimeras	32% 4DIV;<br>15.8% 5DIV	0.8 (4DIV/overall chimeras);<br>0.395 (5DIV/overall chimeras)	Masaki et al., 2015
	15.6% (7/45) fetal	--	--	Kobayashi et al., 2010
	5.79% (74/1279) neonates	--	--	Isotani et al., 2011
Rat iPSC – Preimplantation mouse embryo	41% neonate	48.25% 4DIV;<br>23.75 % 5DIV	1.18 (4DIV/neonate chimera);<br>0.58(5DIV/neonate chimera)	Masaki et al., 2015
	21.1% (23/109) fetal	--	--	Kobayashi et al., 2010
Rat iPSC-Preimplantation rat embryo	63.3 (19/30) fetal	--	--	Kobayashi et al., 2010
Pig iPSC-Preimplantation pig embryo	85.3% (29/34) neonate			West et al., 2010;<br>West et al., 2011
Monkey ESC - Preimplantation monkey embryo	0% (0/7) fetuses at mid-gestation after injection into monkey blastocysts	--	--	Tachibana et al., 2012
Monkey ESC - Preimplantation mouse embryo	0% (0/9) embryos at E8.5 (fig S6)	5.25% 4DIV;<br>0% 5 DIV	0 (5DIV/ fetal chimera)	Masaki et al., 2015
Region-selective monkey ESCs – Post-implantation mouse embryo	--	52.9% (18/34) embryos injected at E7.5	--	Wu et al., 2015
Naïve monkey iPSC – Preimplantation mouse embryo	5% (8/160) 8 cell & blastocysts injected, 6 at E10.5; 2 at E16	--	--	Fang et al., 2014

Naïve monkey iPSC – Preimplantation monkey embryo	14.3% (2/14 fetal)<br><br>63.6% (21/33) resulting blastocysts were GFP+ from 59 monkey embryos injected with CES-l naïve iPSCs	80% 14 monkey embryos were injected with 3-12 CES line naïve iPSCs, of which 4/5 resulting blastocysts were GFP+;<br>51.8% 2DIV 57 GFP+ blastocysts of 110 injected	3.62 2DIV/fetal chimeras	Chen et al., 2015
Monkey iPSC - Preimplantation mouse embryo	0% (0/104) embryos injected with primed iPSCs, 0 were + of 50 recovered at E10.5 and E16	--	--	Fang et al., 2014
Reset human iPSCs - Preimplantation mouse embryo	--	8 cell aggregation + 48h: 0% (0/37) PB-Cherry+ conventional hPSCs; 14.3% (6/42) PB-Cherry+ reset hPSCs;<br>16.3% (8/49) GFP+ reset hPSCs.<br>Blastocyst injection + 72h: 0% (0/17) GFP+ conventional hPSCs;<br>28.1% (9/32) GFP+ reset hPSCs.<br>No contribution of reset hPSCs detected in further culture.	--	Takashima et al., 2014
	--	9.33% 4DIV;<br>0% 5DIV	--	Masaki et al., 2015
Naïve human ESC - Preimplantation mouse embryo	6.19%, (13/210) GFP+ E8.5-E10.5 mid-gestation chimeras from recovered morulae/blastocyst	~3% GFP+ blastocysts 24-36h after injection of primed hPSCs into mouse morulae; ~78% GFP+ after injection of naïve hPSCs (estimated from Extended Data Fig 10b).	0.48 (1-1.5DIV/ fetal chimera)	Gafni et al., 2013
	0% 5i/L/FA hPSCs (0/368) when recovered at E9.5-10.5;<br>0% NHSM culture condition hPSCs (0/195) when	--	--	Theunissen et al., 2014

	recovered at E9.5-10.5			
	0.9% 4i/L/A (6/660) isolated at E9.5-12.5; 0.26% 5i/L/A (1/139) isolated at E9.5-12.5; 0% 6i/L/A (0/224) isolated at E9.5-12.5; 0% t2i/L/DOX+RI (0/246) isolated at E9.5-12.5; 0% NHSM (0/119) isolated at E9.5-12.5	--	--	Theunissen et al., 2016
Human ESCs - Preimplantation mouse embryo	4.17% (1/24) normal positive embryos at E8.5, 16.67% (4/24) total positive embryos at E8.5	39% of blastocysts contain hESCs 2DIV after morula aggregation	9.35 (2DIV/ normal fetal chimera)	James et al., 2006
Human ESCs - Post-implantation mouse embryo	--	99.2% (59/60) embryos injected at E6.5 or E7.5	--	Mascetti and Pedersen, 2016
	--	25% (8/32) embryos injected at E7.5; all OCT4 +, thus undifferentiated, non chimeric	--	Wu et al., 2015
Region-selective Human ESCs – Postimplantation mouse embryo	--	60.69% (45/56) embryos injected at E7.5	--	Wu et al., 2015
Human iPSC - Preimplantation mouse embryo	--	8.4% 4DIV; 0% 5DIV	--	Masaki et al., 2015
DR-human IPSC (dissociation-resistant) - Preimplantation mouse embryo	--	18% 4DIV; 6.6% 5DIV; 0% 6DIV	--	Masaki et al., 2015
Human iPSCs – Postimplantation mouse embryo	--	71.8% (128/179) embryos injected at E6.5 or E7.5	--	Mascetti and Pedersen, 2016

Table S1. Summary of pluripotent stem cell chimera rates

Chimeric contribution from diverse donor cell types in fetal and full term and in vitro chimera assays. Table S1 summarizes chimera contributions for mouse, rat, pig, monkey, and human pluripotent stem cells (embryonic stem cells, ESCs; induced pluripotent stem cells, iPSCs; and epiblast stem cells, EpiSCs). Outcomes shown include fetal and full term in vivo chimera rate; in vitro chimera rate (outgrowth assay, showing data for successive days in vitro, DIV); and the ratio of their incidence, the "chimeric index" (in vitro/ in vivo chimera rate, where full term and fetal rates are averaged as overall chimeras) is shown where this could be calculated. Outcomes of PSC transplantation to post-implantation mouse embryos are shown as overall average percentage chimerism for each study cited.

Supplemental References

Bao, S., Tang, F., Li, X., Hayashi, K., Gillich, A., Lao, K., and Surani, M.A. (2009). Epigenetic reversion of post-implantation epiblast to pluripotent embryonic stem cells. Nature *461*, 1292–1295.

Okita, K., Ichisaka, T., and Yamanaka, S. (2007). Generation of germline-competent induced pluripotent stem cells. Nature *448*, 313–317.
